# Review of the Chinese species of the genus *Varma* Distant (Hemiptera, Fulgoromorpha, Tropiduchidae), with description of two new species

**DOI:** 10.3897/zookeys.417.6798

**Published:** 2014-06-18

**Authors:** Zhi-Min Chang, Zheng-Guang Zhang, Lin Yang, Xiang-Sheng Chen

**Affiliations:** 1 Institute of Entomology / Special Key Laboratory for Developing and Utilizing of Insect Resources, Guizhou University, Guiyang, Guizhou, 550025, P.R. China; 2 School of Life Sciences, Jinggangshan University, Ji’an, Jiangxi, 343009, P.R. China; 3 College of Animal Science, Guizhou University, Guiyang, Guizhou, 550025, P.R. China

**Keywords:** Fulgoroidea, morphology, taxonomy, distribution

## Abstract

Two new species of *Varma* Distant, 1906, *V. falcata* Chang & Chen, **sp. n.** (China: Guizhou) and *V. lobata* Chang & Chen, **sp. n.** (China: Guizhou) are described and illustrated. The female genitalia of four speices including two known species are described and illustrated for the first time. The diagnostic characters of this genus are redefined. A checklist to the species of *Varma* in China is given. The Keys on male and female genitalia to the Chinese species of *Varma* are provided.

## Introduction

The tropiduchid genus *Varma* was established by Distant (1906) with *Serida fervens* Walker, 1857 from Borneo as its type species. He described the second species *Varma tridens* from Sri Lanka. Then Distant (1909) described one species *Varma obliqua* in this genus. Melichar (1914) transferred the genus *Varma* into Tropiduchidae and placed it in the tribe Tropiduchini, added one species *Varma distanti* from India. Metcalf (1954) and Fennah (1982) recognised the treatment. In China, as the first record of the genus *Varma*, two species *Varma gibbosa* and *Varma bimaculata* were described by Wang and Liang (2008). Then Men et al. (2010) added one species *Varma serrata* from China. Up to now, seven species have been reported worldwide, of three species recorded in southwestern China.

Much attention were paid to the genus *Varma*, however, little information has been reported on female genitalia of the genus, especially in species identification. In this paper, two new species *Varma falcata* Chang & Chen, sp. n. from Guizhou (China) and *Varma lobata* Chang & Chen, sp. n. from Yunnan (China) are described and illustrated. The female genitalia of four speices including two known species, *Varma gibbosa* and *Varma serrata* are described and illustrated for the first time, as useful characters for species identification. The diagnostic characters of this genus are redefined. A checklist to the species of *Varma* in China is given. The Keys on male and female genitalia to the Chinese species of *Varma* are provided.

## Material and methods

External morphology was observed under a stereoscopic microscope and dimensions of characters were measured with an ocular micrometer. Measurements are given in millimeters (mm). Abdomens were removed and macerated in 10% KOH overnight, washed in water and then removed to glycerine. Observations and drawings were done under a Leica MZ 12.5 stereomicroscope. Illustrations were scanned with Canon CanoScan LIDE 100 and imported into Adobe Photoshop 8.0 for labeling and plating composition. Photographs of the types were taken with a KEYENCE VHX-1000C. The type specimens are deposited in the Institute of Entomology, Guizhou University, Guiyang, China (GUGC).

Morphological terminology follows that of Bourgoin and Huang (1990) and Bourgoin (1993) for male and female genitalia.

## Taxonomy

### Genus Varma

Taxon classificationAnimaliaHemipteraTropiduchidae

Distant, 1906

Varma Distant, 1906: 330; Distant 1909: 171; Melichar 1914: 117; Wang and Liang 2008: 116.

#### Type species.

*Serida fervens* Walker, 1857, by original designation.

#### Diagnosis.

**Figures 1–4.**
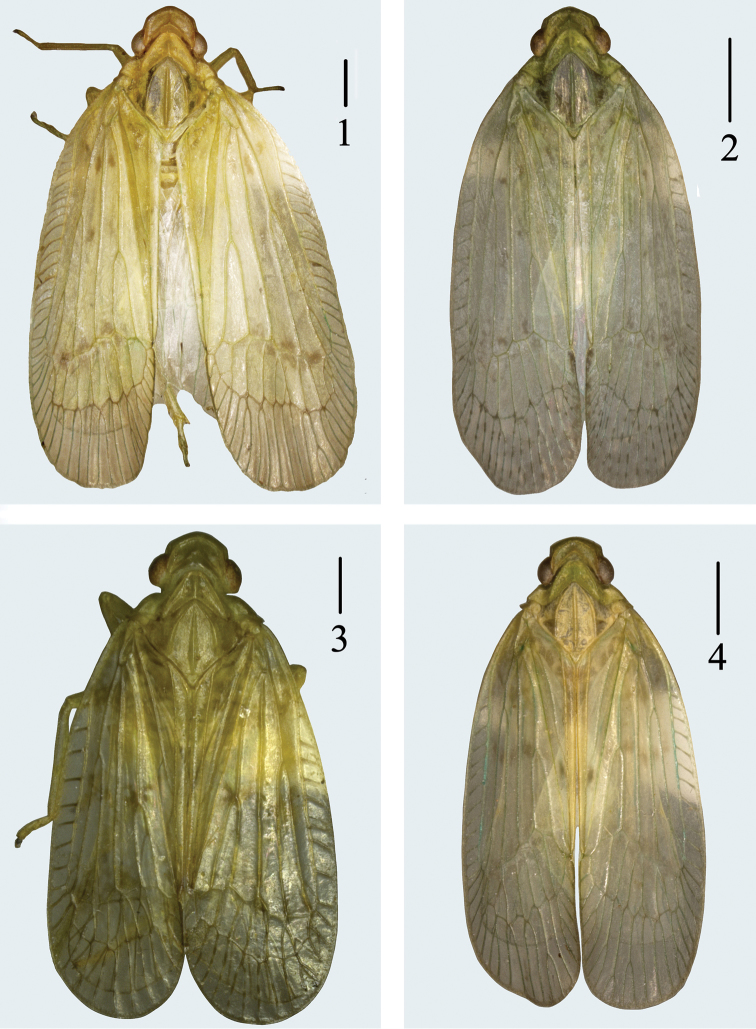
Dorsal habitus of *Varma* species. **1** *Varma falcata* Chang & Chen, sp. n.; **2** *Varma lobata* Chang & Chen, sp. n.; **3** *Varma gibbosa* Wang & Liang; **4** *Varma serrata* Men & Qin. Scale bars = 1.0 mm.

**Figures 5–15.**
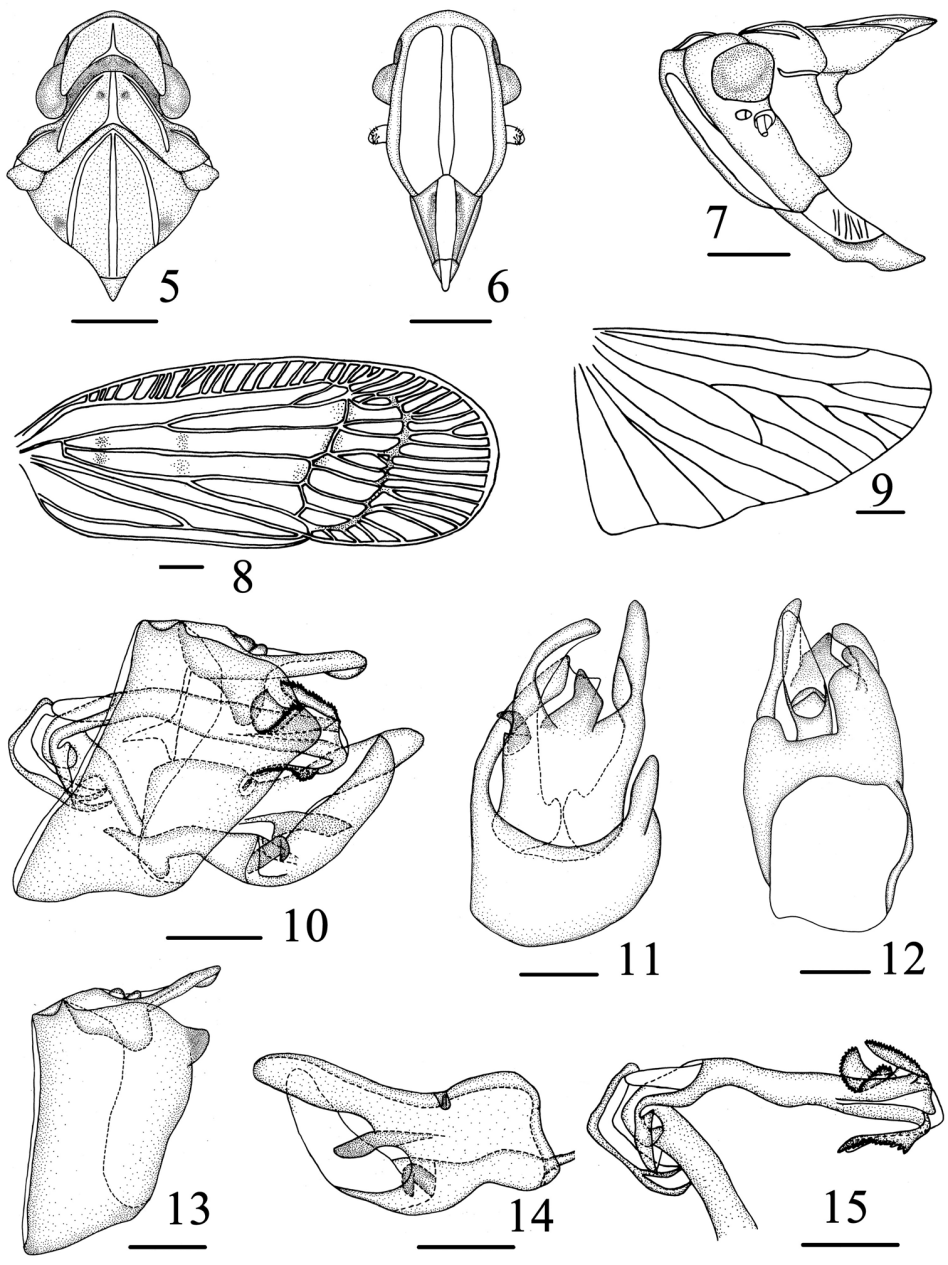
*Varma falcata* Chang & Chen, sp. n. **5** Head and thorax, dorsal view **6** Head, ventral view **7** Head and thorax, lateral view **8** Forewing **9** Hind wing **10** Male genitalia, lateral view **11** Pygofer and gonostyli, ventral view **12** Male genitalia, dorsal view **13** Pygofer and anal segment, left side **14** Gonostyli, right side **15** Aedeagus, left side. Scale bars = 1.0 mm (**5–9**), 0.5 mm (**10–15**).

Body slender, somewhat dorsoventrally depressed. Head (Figs 1–4, 5, 24) produced in front of eyes. Vertex (Figs 1–4, 5, 24) shorter in middle than broad at base (about 2.1–4.8:1), disc depressed, unicarinate carina, not reaching anterior margin. Frons (Figs 6, 25) unicarinate, longer in middle line than wide than broad (about 1.3–1.5:1). Pronotum (Figs 5, 24) tricarinate, wider than long in middle (about 3.1-5.0:1). Mesonotum (Figs 5, 24) tricarinate, wider than long in middle (about 1.1–1.3:1). Hind tibia with 3 lateral spines, spinal formula of hind leg 6(5)-5-2. Forewing (Figs 1–4, 8, 27) subhyaline, about 2.5 times longer than the widest breadth, corium without granulation, costal cell with various oblique transverse veins, nodal line and subapical line distinct. Male genitalia (Figs 10–15, 29–34) with pygofer asymmetrical, irregularly subquadrate, posterior marging produced various lobes; anal tube (urite X) symmetrical, anal styles (paraproct and epiproct) relative small, not surpassing apex of anal tube; gonostyli asymmetrical, with a strong stout process in left side, with a small process in right side; fused basally, with various median processes in ventral view; aedeagus tubular, expanded apex, with several lobes or band processes.

#### Female genitalia.

**Figures 16–23.**
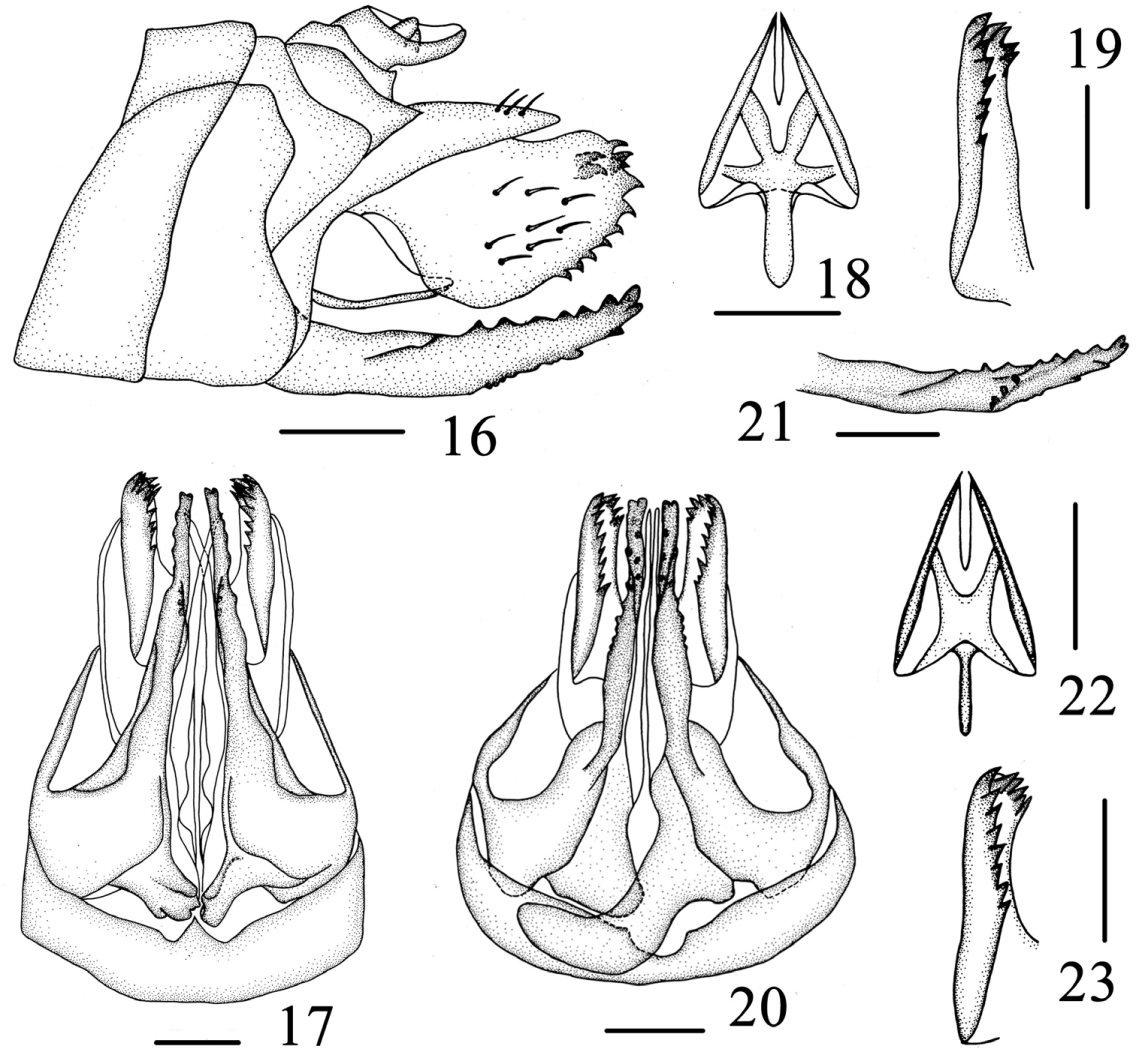
Female genitalia. **16**–**19** *Varma falcata* Chang & Chen, sp. n. **16** Female genitalia, lateral view **17** Female genitalia, ventral view **18** Gonapophyses IX, ventral view **19** Gonoplace, ventral view **20**–**23** *Varma gibbosa* Wang & Liang **20** Female genitalia, ventral view **21** Gonapophyses VIII, lateral view **22** Gonapophyses IX, ventral view **23** Gonoplace, ventral view. Scale bars = 0.5 mm (**16–23**).

Gonapophyses VIII (first valvula) (Figs 16, 21, 35, 40) saw-like, strongly sclerotized, with 6-8 distinct teeth on dorsal margin, ventral margin with about 3-4 distinct teeth. Gonapophyses IX (second valvula) (Figs 18, 22, 35, 37, 41) degradated, triangular. Gonoplace (third valvula) (Figs 19, 23, 38, 42) stout, membranous, formed about 10-14 teeth on ventral margin and apical margin (Figs 19, 23, 38, 42). In ventral view, endogonocoxal lobe (Figs 17, 20, 36, 39) not bilaterally symmetrical, in the base of the gonapophyses VIII produced mesad irregular process. Sternite VII with posterior margin depressed or convex (Figs 17, 20, 36, 39).

**Figures 24–34.**
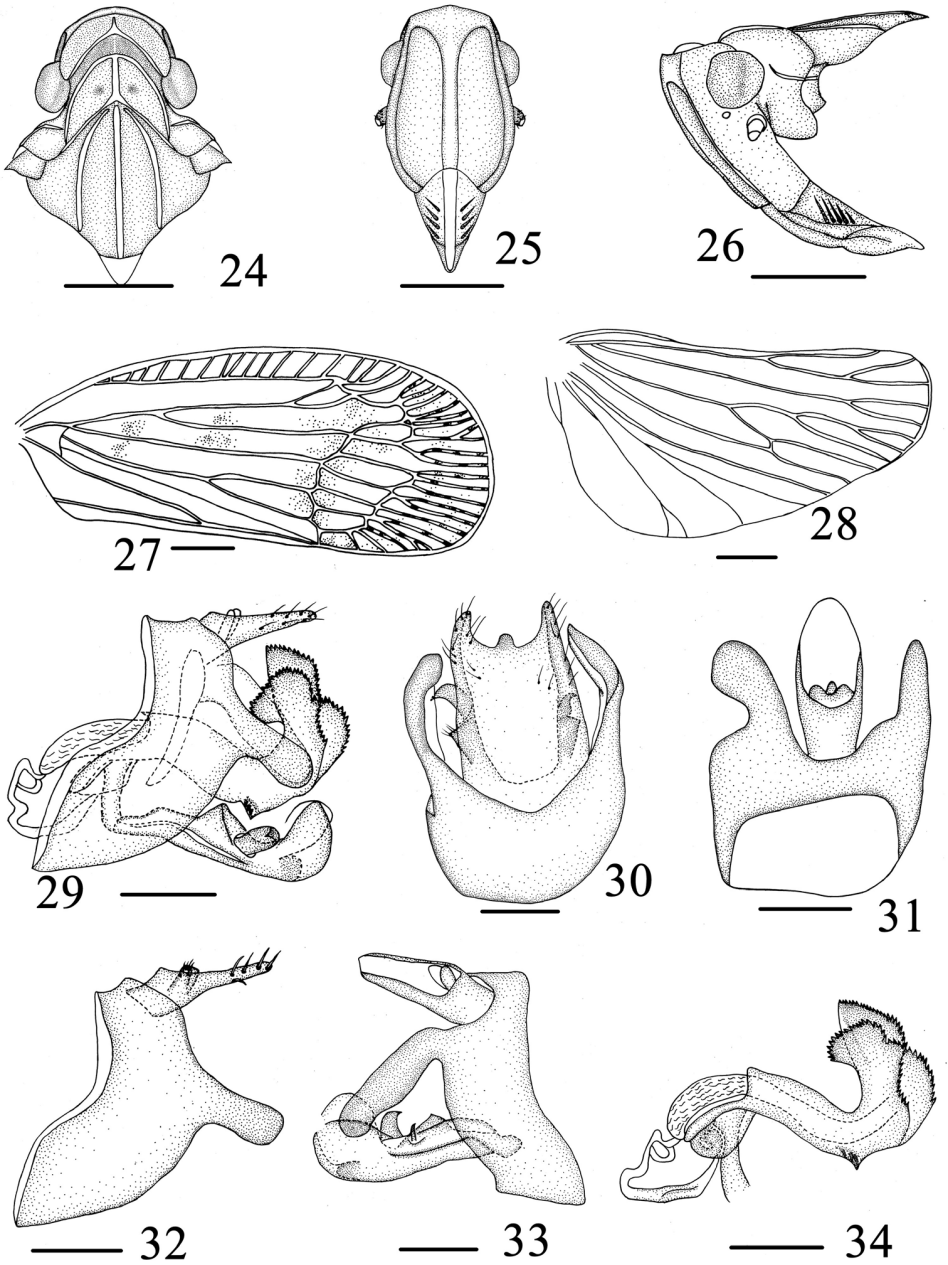
*Varma lobata* Chang & Chen, sp. n. **24** Head and thorax, dorsal view **25** Head, ventral view **26** Head and thorax, lateral view **27** Forewing **28** Hind wing **29** Male genitalia, lateral view **30** Pygofer and gonostyli, ventral view **31** Male genitalia, dorsal view **32** Pygofer and anal segment, left side **33** Male genitalia, right side **34** Aedeagus, left side. Scale bars = 1.0 mm (**24–28**), 0.5 mm (**29–34**).

**Figures 35–42.**
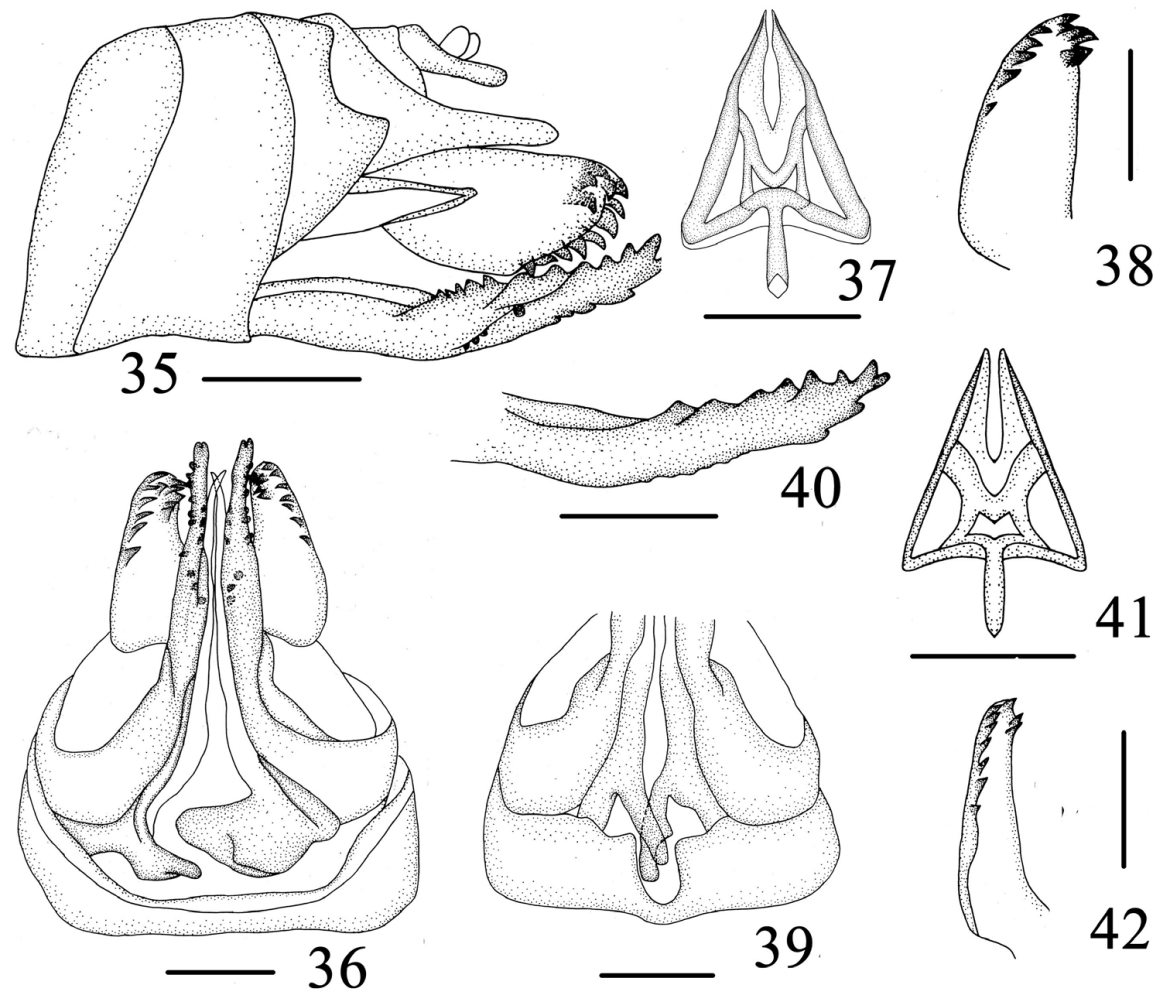
Female genitalia. **35**–**38** *Varma lobata* Chang & Chen, sp. n. **35** Female genitalia, lateral view **36** Female genitalia, ventral view **37** Gonapophyses IX, ventral view **38** Gonoplace, ventral view **39**–**42** *Varma serrata* Men & Qin **39** Female genitalia, lateral view **40** Gonapophyses VIII, lateral view **41** Gonapophyses IX, ventral view **42** Gonoplace, ventral view. Scale bars = 0.5 mm (**35–42**).

#### Distribution.

Oriental region.

#### Checklist of species of *Varma* Distant, 1906 in China

*Varma bimaculata* Wang & Liang, 2008; China (Xizang)

*Varma falcata* Chang & Chen, sp. n.; China (Guizhou)

*Varma gibbosa* Wang &Liang, 2008; China (Xizang)

*Varma lobata* Chang & Chen, sp. n.; China (Yunnan)

*Varma serrata* Men & Qin, 2010; China (Yunnan, Hunan)

#### Keys to species of genus *Varma*, 1906 in China

(based on male genitalia, ♂)

**Table:** 

1	Pygofer with posterior margin produced into a distinct process in right side	2
-	Pygofer without posterior margin produced into a distinct process in right side	3
2	Pygofer with posterior margin produced into a trapezoidal process in right side (see Men and Qin: 263, Fig. 2A, I, J)	*Varma serrata* Men & Qin
-	Pygofer with posterior margin produced into a strip in right side (Figs 29, 32, 33)	*Varma lobata* Chang & Chen, sp. n.
3	Gonostyli with subcircular or subglobose lobe at apical inner margin	4
-	Gonostyli with a falcate lobe at apical inner margin (Fig. 11)	*Varma falcata* Chang & Chen, sp. n.
4	Aedeagus with apical part expanded into two hemispherical protuberances, curved through about 180 degree (see Wang and Liang: 118, Figs 8, 9)	*Varma gibbosa* Wang & Liang
-	Aedeagus with apical part expanded into a hemispherical protuberance, curved through about 90 degree, then extended into an irregularly contorted scoop-shape plate (see Wang and Liang: 121, Figs 21, 22)	*Varma bimaculata* Wang & Liang

(based on female genitalia,♀)

**Table:** 

1	Sternite VII with posterior margin project triangularly in middle; endogonocoxal lobe produced mesad irregular trapeziums, more broader in left side, more slender in right side (Fig. 17)	*Varma falcata* Chang & Chen, sp. n.
-	Sternite VII with posterior margin formed a shallow pit in middle	2
2	Sternite VII with posterior margin formed a pit in middle about1/2; endogonocoxal lobe produced mesad irregular triangle, more narrow in left side, more broader in right side (Fig. 36)	*Varma lobata* Chang & Chen, sp. n.
-	Sternite VII with posterior margin formed a pit in middle less than1/2	3
3	Sternite VII with posterior margin formed a narrow and deep pit in middle 1/9; endogonocoxal lobe produced mesad different rods (Fig. 39)	*Varma serrata* Men & Qin
-	Sternite VII with posterior margin formed a shallow pit in middle 1/7; endogonocoxal lobe produced mesad irregular protuberances, triangular in left side, falculate in right side (Fig. 20)	*Varma gibbosa* Wang & Liang

Note: this key didn’t refer to *Varma bimaculata* Wang & Liang, as we didn’t have the female specimens.

### Varma bimaculata

Taxon classificationAnimaliaHemipteraTropiduchidae

Wang & Liang, 2008

Varma bimaculata Wang & Liang, 2008: 120.

#### Material examined.

No specimen has been collected by the authors.

#### Distribution.

China (Xizang).

### Varma falcata

Taxon classificationAnimaliaHemipteraTropiduchidae

Chang & Chen sp. n.

http://zoobank.org/37F52044-C2AD-47A3-8512-D810C1F31D28

Figs 1 , 5–15

#### Type material.

Holotype: ♂, **China:** Guizhou, Dushan, Duliujiangyuan Wetlands (25°50'N, 107°32'E), 12 July 2012, Q.-Z. Song; paratypes: 1 ♂, 4 ♀♀, same data as holotype; 2 ♀♀, Guizhou, Fanjingshan National Nature Reserve (27°44'N, 109°13'E), 22-24 Sept. 2011, X.-F. Yu and Z.-H. Fan; 1 ♀, Fanjingshan National Nature Reserve, 1 June 2002, X.-S. Chen; 1 ♀, Leigongshan National Nature Reserve (26°23'N, 108°04'E), 10 July 2011, Z.-M. Chang.

#### Description.

Body length (from apex of vertex to tip of forewings): male 12.1–12.8 mm (N = 2), female 13.0–13.5 mm (N = 4).

**Coloration.** General colour yellowish green to stramineous yellow. Head and pronotum pale green to pale tawny. Mesonotum pale green or pale ocherous. Abdomen tawny. Forewings pale yellowish green, basal part with two light brownish spots, around nodal line with light brownish fasciae.

**Head and thorax.** Vertex (Fig. 5) broader than long in middle line (2.4:1). Frons (Fig. 6) obviously longer in middle than widest breadth (1.4:1), widest at apical fourth, anterior margin and posterior arch, median carina broad and ridged. Pronotum (Fig. 5) obviously wider than long in middle (3.8:1). Mesonotum (Fig. 5) wider than long in middle (1.1:1). Forewing about 2.4 times longer than the widest, venations as in Fig. 8. Hind wing with venation as in Fig. 9. Spinal formula of hind leg 6-5-2.

**Male genitalia.** Male genitalia (Figs 10–14) with pygofer not bilaterally symmetrical; in proflile left side, irregularly subquadrate, dorsal margins and ventral margin subparallel, anterior margin and posterior margin nearly subparallel, posterior margin near its upper end produced into a short and indistinct lobe; in right side, dorsal margin incline to venter, anterior margin near dorsal 1/3 concave, lateral margins subparallel. Anal tube (urite X) (Figs 12, 13) symmetrical and long, surpassing apex of aedeagus in lateral view, with anal styles short, not surpassing apex of anal tube. In ventral view, gonostyli (Figs 10–12, 14) with basal 2/3 fused together, medially produced into a falcate lobe. Aedeagus (Fig. 15) tubular, relatively long, narrow at base and expanded apex, with three processes, of one band process at lateral ventral margin, of one lobe in doral margin, directed anteriorly, of one flexural waviness on the right side, all procrsses with serrated margins. Periandrium fissure-like, exposed at the apex. Corpus connectivi stout, rod-like.

**Female genitalia.** Gonapophyses VIII (first valvula) (Fig. 16) saw-like, strongly sclerotized with 6-7 distinct teeth on dorsal margin, ventral margin with about 4 distinct teeth. Gonapophyses IX (second valvula) (Fig. 18) degradated, triangular. Gonoplace (third valvula) (Fig. 19) stout, membranous, formed 12 teeth on ventral margin and apical margin. In ventral view, endogonocoxal lobe (Fig. 17) in the base of the gonapophyses VIII produced mesad irregular trapeziums, that of left side more broader, paw-like, with 2 small teeth, that of right more slender, lateral margin dented. Sternite VII (Fig. 17) with posterior margin project triangularly in middle.

#### Etymology.

The new species is named after the presence of a falcate process at apically inner margin of gonostyli.

#### Distribution.

China (Guizhou).

#### Remarks.

This new species is similar to *Varma serrata* Men & Qin, 2010 in external appearance, but can be distinguished from the latter in the gonostyli with a falcate lobe at apically inner margin (with semicircular lobe in *serrata*) (Fig. 11); posterior margin of pygofer without lobe process near its upper end on right side (with posterior margin with trapezoidal lobe directed caudoventrad on right side in *serrata*) (Figs 10, 13); apical part of the aedeagus bearing one band process at lateral ventral margin, with one lobe in doral margin, on the right side of the aedeagal shaft with one flexural waviness (in *serrata*, with apical part of aedeagus bearing a semicircular ribbon-like plate at left side side, on the right side of the aedeagal shaft with a pediform flate plate and wing-shaped lobe) (Fig. 15).

### Varma gibbosa

Taxon classificationAnimaliaHemipteraTropiduchidae

Wang & Liang, 2008

Figs 3 , 20–23

Varma gibbosa Wang & Liang, 2008: 117.

#### Material examined.

2 ♂♂, 1 ♀, **China**, Xizang, 1960m, 22 Aug. 2005, Z.-H. Yang.

#### Distribution.

China (Xizang).

#### Female genitalia.

Gonapophyses VIII (first valvula) (Fig. 21) saw-like, strongly sclerotized with 7-8 distinct teeth on dorsal margin, ventral margin with 3-4 distinct teeth. Gonapophyses IX (second valvula) (Fig. 22) degradated, triangular. Gonoplace (third valvula) (Fig. 23) stout, membranous, formed 13 or 14 teeth on ventral margin and apical margin. In ventral view, endogonocoxal lobe (Fig. 20) in the base of the gonapophyses VIII produced mesad not bilateralll symmetrical, that of left triangular, apex obtuse, that of right falculate. Sternite VII (Fig. 20) with posterior margin forming a shallow pit in middle 1/7.

#### Remarks.

For the female species, Sternite VII with posterior margin of *Varma gibbosa* is similar to *Varma serrata*, but can be from the latter in the pit of posterior margin more shallow and relative more broader (more deep and narrow in *serrata*), left of endogonocoxal lobe triangular, apex obtuse, that of right falculate (that of left thin rods, that of right slender, with one triangular protrusion in the outer edge, in *serrata*) (Figs 20, 39).

### Varma lobata

Taxon classificationAnimaliaHemipteraTropiduchidae

Chang & Chen sp. n.

http://zoobank.org/1A64F0A0-1905-4E63-AFCB-46DFDD89C124

Figs 2 , 24–34

#### Type material.

Holotype: ♂, **China:** Yunnan, Jinping Fenshuiling Native Nature Reserve (22°46'N, 103°14'E), 7 July 2012, W.-B. Zheng; paratypes: 4 ♂♂, 6 ♀♀, same data as holotype.

#### Description.

Body length (from apex of vertex to tip of forewings): male 9.2–9.5 mm (N = 6), female 11.1–11.8 mm (N = 4).

**Coloration.** General color pale green to greenish-yellow. Vertex, pronotum, mesonotum pale green. Forewings light green, marked with irregular brown spots at the base and middle and around nodal line, with 3-4 ranks of fine speckles and suffused fuscous between apical margin and the first subapical line.

**Head and thorax.** Vertex (Fig. 24) distinctly broader than long in middle line (4.8:1). Frons (Fig. 25) longer in middle than widest breadth (1.3:1), widest at apical fourth. Pronotum (Fig. 24) obviously wider than long in middle (4.5:1). Mesonotum (Fig. 24) wider than long in middle (1.2:1). Forewing about 2.4 times longer than the widest, venations as in Fig. 27. Hind wing with venation as in Fig. 28. Spinal formula of hind leg 6 (5)-5-2.

**Male genitalia.** Male genitalia (Figs 29–34) with pygofer not bilaterally symmetrical, in proflile left view, irregularly stomach-like, posterior margin produced caudad with a lobe near its upper end; in right side, irregularly subquadrate, posterior margin producrd into strip directed caudoventrad (Figs 29, 31, 32). Anal tube (urite X) (Figs 31, 32) symmetrical, not surpassing apex of aedeagus in lateral view, anal styles relatively small, not surpassing apex of anal tube. In ventral view, gonostyli (Figs 29, 30, 33) with basal 3/4 fused together, medially produced into sub-semicircular lobe. Aedeagus (Figs 29, 34) tubular, relatively long, narrow at base and distinctly expanded apex, splitting into two lobe plates, directed into dorsal margins, both with serrated margins, of one prongy in left view, of one sub-oblong lobe in right side, with 3-4 denticulate structure in ventral margins near basal 2/3. Periandrium fissure-like, exposed in middle. Corpus connectivi stout, rod-like.

**Female genitalia.** Gonapophyses VIII (first valvula) (Fig. 35) saw-like, strongly sclerotized with 7 distinct teeth on dorsal margin, ventral margin with 3-4 distinct teeth. Gonapophyses IX (second valvula) (Fig. 37) degradated, triangular. Gonoplace (third valvula) (Fig. 38) stout, membranous, formed 12 or 13 teeth on ventral margin and apical margin. In ventral view, endogonocoxal lobe (Fig. 36) in the base of the gonapophyses VIII produced mesad irregular triangle, that of left more narrow, that of right more broader. Sternite VII (Fig. 36) with posterior margin forming a pit in middle 1/2.

#### Etymology.

The name of the new species results from apex of aedeagus, splitted into two lobe plates.

#### Distribution.

China (Yunnan).

#### Remarks.

This new species can be distinguished from other species in the genus by the following combination of characters: (1) Gonostyli with a sub-semicircular lobe at apically inner margin (Fig. 30). (2) Apex of aedeagus, splitted into two lobe plates, directed into dorsal margins, of one sub-oblong lobe, of one sub-oblong lobe, with 3-4 denticulate structure in ventral margins near basal 2/3 (Fig. 34). (3) Pygofer, irregularly stomach-like in proflile left view; right side, posterior margin producrd into a strip (Figs 29, 32). (4) Sternite VII with posterior margin forming a pit in middle 1/2 (Fig. 36).

### Varma serrata

Taxon classificationAnimaliaHemipteraTropiduchidae

Men & Qin, 2010

Figs 4 , 39–42

Varma serrata Men & Qin, 2010: 116.

#### Material examined.

2 ♂♂, 3 ♀♀, **China**, Yunnan, Lushui, 1960m, 14 Aug. 2006, Q.-Z. Song; 2 ♀♀, Hunan, Langshan, 5 Oct. 2010, X.-S. Chen; 2 ♂♂, Yunnan, Lushui, 21 July 2012, J.-K. Long.

#### Female genitalia.

Gonapophyses VIII (first valvula) (Fig. 40) saw-like, strongly sclerotized with 7 distinct teeth on dorsal margin, ventral margin with 3 distinct teeth. Gonapophyses IX (second valvula) (Fig. 41) degradated, triangular. Gonoplace (third valvula) (Fig. 42) stout, membranous, formed 10 teeth on ventral margin and apical margin. In ventral view, endogonocoxal lobe (Fig. 39) in the base of the gonapophyses VIII produced mesad not bilateralll symmetrical, that of left thin rods, that of right slender, with one triangular protrusion in the outer edge, Sternite VII with posterior margin formed a narrow and deep pit in middle1/9 (Fig. 39).

#### Distribution.

China (Yunnan, Hunan).

#### Remarks.

The author examined many species of *Varma serrata*, and then found that the forewings marked with very light irregular brown spots at the base and middle and around nodal line (Fig. 4).

### Geographic distribution of *Varma* worldwide

According to the geographic distribution map, all speices distributed in Oriental region.

**Figure 43.**
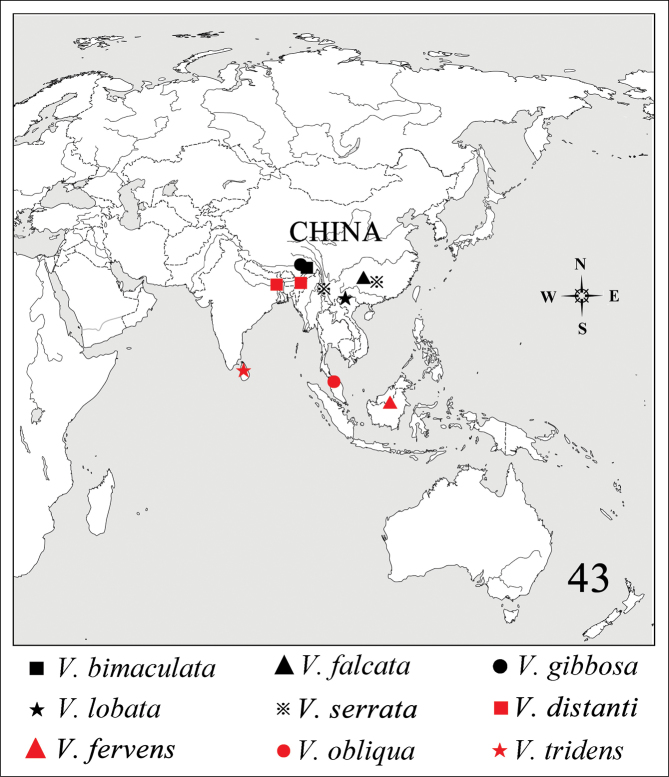
Geographic distribution of *Varma* worldwide

## Discussion

The female genitalia were generally used for higher taxon in the Tropiduchidae family, such Fennah (1982) as important tribes’ characters. However, few characters were used in species identification for the instability of the female genitalia, such as the numbers of teeth of gonapophyses VIII and gonoplace. Fortunately, we found that there are steady specific differences in the endogonocoxal lobe and sternite VII in genus *Varma* (Figs 17, 20, 36, 39). These characters were used to record new species in *Leptovanua* Melichar, 1914 (Tropiduchini), *Neocatara* Distant, 1910 (Tropiduchini) (Fennah 1970). It seems unclear that these characters are suited to others genus in the tribeTropiduchini for inadequate specimens.

## Supplementary Material

XML Treatment for Varma

XML Treatment for Varma bimaculata

XML Treatment for Varma falcata

XML Treatment for Varma gibbosa

XML Treatment for Varma lobata

XML Treatment for Varma serrata
